# Cytochrome P450 Genes Mediate High-Temperature Adaptation Under Diverging Humidity Conditions in *Tuta absoluta*

**DOI:** 10.3390/ijms27072935

**Published:** 2026-03-24

**Authors:** Hina Gul, Guru-Pirasanna-Pandi Govindharaj, Ghulam Murtaza, Farman Ullah, Jun Huang, Wenchao Guo, Raul Narciso C. Guedes, Nicolas Desneux, Xiaowei Li, Yaobin Lu

**Affiliations:** 1 State Key Laboratory for Quality and Safety of Agro-Products, Key Laboratory of Biotechnology in Plant Protection of MOA of China and Zhejiang Province, Institute of Plant Protection and Microbiology, Zhejiang Academy of Agricultural Sciences, Hangzhou 310021, China; gulhina680@gmail.com (H.G.); farmanullah787@gmail.com (F.U.); junhuang1981@126.com (J.H.); 2 Plant Quarantine Division, ICAR-National Bureau of Plant Genetic Resources, Regional Station, Hyderabad 500030, India; guruagri@gmail.com; 3 State Key Laboratory of Plant Diversity and Specialty Crops, South China Botanical Garden, Chinese Academy of Sciences, Guangzhou 510650, China; murtazabwn54@gmail.com; 4 Institute of Bio-Interaction, Xianghu Laboratory, Hangzhou 311258, China; 5 Institute of Plant Protection, Xinjiang Uygur Autonomous Region Academy of Agricultural Sciences, Key Laboratory of Integrated Pest Management on Crops in Northwestern Oasis, Ministry of Agriculture and Rural Affairs, Xinjiang Key Laboratory of Agricultural Biosafety, Urumqi 830091, China; gwc1966@163.com; 6 Departamento de Entomologia, da Federal de Viçosa, Viçosa 36570-900, MG, Brazil; guedes@ufv.br; 7 UMR ISA, INRAE, Université Côte d’Azur, 06000 Nice, France; nicolas.desneux@inrae.fr

**Keywords:** RNA interference, cytochrome P450, abiotic stress, thermal adaptation, humidity stress, nanocarriers

## Abstract

Temperature and humidity are critical abiotic factors shaping the survival and adaptation of insect pests. However, the molecular mechanisms underlying high-temperature tolerance under contrasting humidity conditions remain poorly understood, particularly in globally invasive species such as the tomato pinworm, *Tuta absoluta.* Previous studies have examined individual stressors, leaving interactive thermo-hygrometric effects on gene expression and survival insufficiently resolved. Here, we assessed the contribution of cytochrome P450 genes to thermal adaptation under low- and high-humidity conditions using transcriptome profiling combined with nanocarrier-mediated RNA interference (RNAi). Third-instar larvae were exposed to high temperature under low humidity (HT-LH: 40 °C, 50% RH) or high humidity (HT-HH: 40 °C, 75% RH) for eight hours. Survival declined from 97.5% in the control to 74.16% under HT-LH and 68.33% under HT-HH conditions. Transcriptome analysis revealed extensive differential gene expression, with 464 genes upregulated and 565 downregulated in HT-LH, and 1145 upregulated and 1166 downregulated in HT-HH. Functional annotation highlighted pathways linked to metabolic regulation, proteostasis, and detoxification, including multiple cytochrome P450-associated processes. RT-qPCR confirmed the upregulation (3–5 fold) of four P450 genes (*CYP6AB327*, *CYP6ABF1b*, *CYP6AE214*, and *CYP9A306c*) under high temperature across both humidity regimes. RNAi-mediated silencing of these genes significantly reduced larval survival, demonstrating their functional role in thermal-hygrometric stress tolerance across. Cytochrome P450 genes underpin the adaptive capacity of the tomato pinworm to high-temperature stress across contrasting humidity conditions, highlighting RNAi-based disruption of P450 function as a promising avenue for sustainable pest management under climate change scenarios.

## 1. Introduction

Climate change is an ongoing global process in which rising temperatures interact with other environmental drivers, notably atmospheric humidity, to shape biological responses with complex and often unpredictable consequences [1,2]. Beyond gradual warming trends, the increasing frequency of abrupt temperature extremes and their interaction with humidity has emerged as a major ecological concern [3]. These combined stressors can profoundly affect organismal physiology, performance, and survival across taxa, including both native and introduced species [4,5,6]. For invasive organisms, the capacity to tolerate and rapidly adapt to novel thermo-hygrometric regimes is a key determinant of establishment success, spread, and eventual economic impact [7,8].

The South American tomato pinworm, *Tuta absoluta* (Meyrick) (Lepidoptera: Gelechiidae), provides a compelling model for examining adaptive responses to interacting abiotic stressors. Native to South America, this species has become one of the most destructive invasive agricultural pests worldwide, causing severe yield losses and posing a major threat to global crop production [9,10,11]. Following its invasion, the tomato pinworm rapidly expanded across Europe, Africa, and Asia, demonstrating an exceptional capacity to establish itself under diverse climatic conditions [10].

Infestations by the tomato pinworm result in extensive damage to tomato crops under both open-field and greenhouse systems, with yield losses reaching 80 to 100% in some regions [12]. The species was first detected in China in 2017 and subsequently spread at an alarming rate, establishing stable populations in more than ten provinces within a short timeframe [13]. Although tomato is its primary host, the pinworm also attacks other economically important solanaceous crops, including potato, brinjal, tobacco, and pepper, further amplifying its agricultural impact [10,14,15]. This broad host range, coupled with aggressive colonization ability, has positioned the tomato pinworm as a persistent constraint to agricultural productivity and food security on a global scale.

The ecological success of the tomato pinworm across contrasting climatic zones is widely attributed to its high physiological plasticity and adaptive molecular mechanisms [16,17]. Temperature and relative humidity are known to exert strong effects on insect development, fecundity, survival, and population dynamics, ultimately shaping invasion trajectories in novel environments [18]. Understanding the genetic basis of thermo-hygrometric adaptation is therefore essential for elucidating how this species tolerates environmental stress, expands its geographical range, and maintains year-round infestations [19,20,21].

Among the gene families implicated in environmental adaptation, cytochrome P450 monooxygenases (P450 genes) play a central role in mediating insect responses to abiotic stressors, including fluctuations in temperature and humidity [22]. P450 enzymes are integral components of detoxification and metabolic pathways, facilitating the biotransformation of endogenous and exogenous compounds and contributing to stress tolerance under adverse environmental conditions [23,24]. Recent studies suggest that P450 genes participate in thermal acclimation by regulating oxidative stress, energy metabolism, and hormonal homeostasis, thereby enhancing insect survival under temperature extremes [25,26]. In addition, P450-mediated metabolic processes may influence cuticular composition and water balance, indirectly supporting tolerance to humidity-related stress [27,28].

Advances in molecular biology have enabled the exploration of previously inaccessible aspects of insect physiology. Among these, RNA interference (RNAi) has emerged as a powerful reverse-genetics tool for functional gene analysis and holds considerable promise for pest management applications [29]. However, RNAi efficiency is strongly influenced by dsRNA delivery methods. In lepidopteran insects, systemic RNAi responses are often limited by rapid dsRNA degradation and inefficient cellular uptake. Recent developments in nanotechnology have begun to overcome these constraints.

Nanocarriers are increasingly employed to protect dsRNA from degradation, enhance targeted delivery, and improve cellular internalization across diverse insect taxa [30]. Encapsulation of dsRNA within nanocarriers improves molecular stability and cellular interaction, substantially enhancing RNAi efficacy [31]. Earlier workers demonstrated that nanocarrier-mediated RNAi markedly increased gene silencing efficiency in the tomato pinworm [32]. Beyond functional genomics, this approach offers a promising framework for developing environmentally sustainable pest management strategies.

Despite their presumed importance, the specific contribution of P450 genes to thermo-hygrometric adaptation in the tomato pinworm remains poorly understood. In this study, we employed a nanocarrier-mediated RNAi approach to target P450 genes and elucidate their functional role in adaptation to combined temperature and humidity stress. By integrating transcriptomic analysis, targeted gene silencing, and bioassays under controlled stress conditions, we provide direct evidence for the involvement of P450 genes in thermal tolerance and desiccation resistance.

## 2. Results

### 2.1. Survival Under Temperature and Humidity Stress

**Figure 1:**
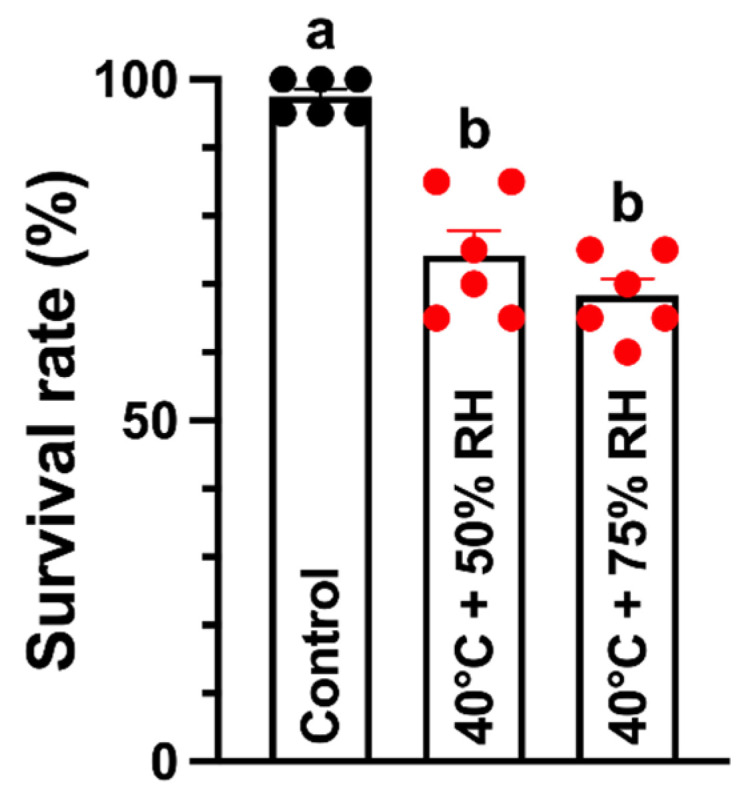
Survival (%) of the tomato pinworm *T. absoluta* following 8 h exposure to high temperature under low humidity (HT-LH: 40 °C, 50% RH) and high humidity (HT-HH: 40 °C, 75% RH). Letters above the bars represent significant differences at *p* < 0.05 level using one-way analysis of variance with Tukey’s post hoc test (IBM, SPSS Statistics, Armonk, NY, USA, version 29).

To evaluate the effects of high temperature combined with contrasting humidity regimes on larval survival, third-instar larvae of the tomato pinworm were exposed for eight hours to high temperature under either low humidity (HT-LH: 40 °C, 50% RH) or high humidity (HT-HH: 40 °C, 75% RH). Larval survival declined significantly under both stress treatments (F_2,17_ = 33.41, *p* < 0.001) relative to the control maintained at 25 °C and 60% RH (Figure 1). Survival decreased from 97.5% in the control to 74.16% under HT-LH and further to 68.33% under HT-HH, indicating that high humidity exacerbated the negative effects of thermal stress.

### 2.2. Transcriptome Assembly, Quality Assessment, and Differential Gene Expression

High-quality transcriptomic datasets were obtained for all treatments (Table 1). Clean reads were in the ranges of 20.46–20.79 million in HT-LH, 19.32–27.70 million in HT-HH, and 21.72–27.87 million in the control group, with consistently high Q30 values (97.94–98.89%). GC content was comparable across treatments (46.05–47.94%), indicating no systematic sequencing bias.

**Figure 2:**
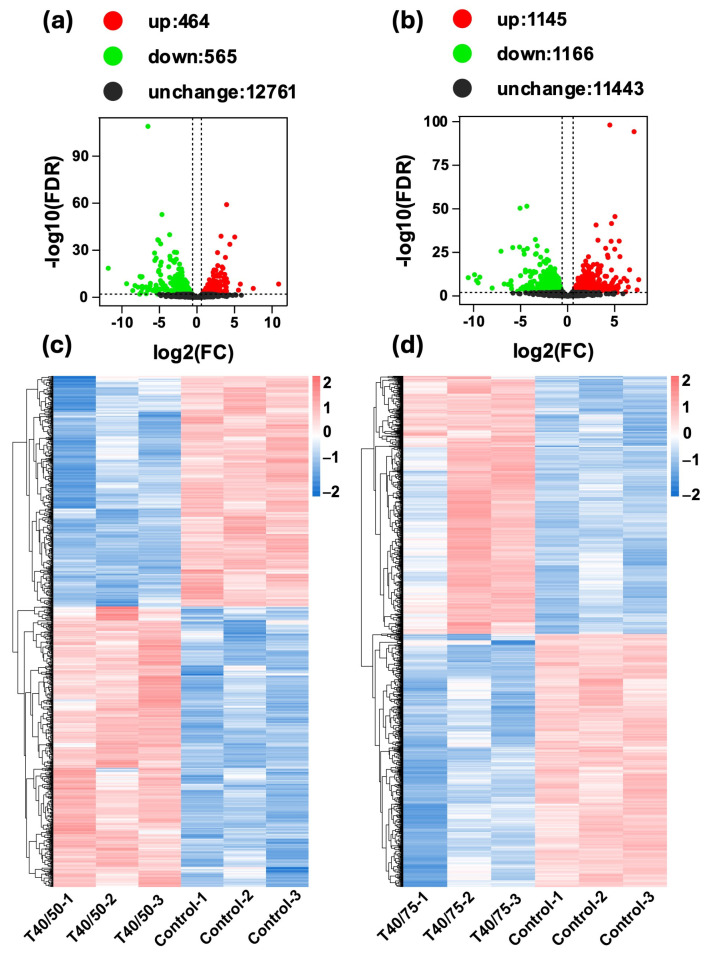
Transcriptomic responses of the tomato pinworm *T. absoluta* to combined temperature and humidity stress. High-throughput RNA-sequencing analysis following 8 h exposure to HT-LH (40 °C, 50% RH) and HT-HH (40 °C, 75% RH): (**a**,**b**) Volcano plots showing differentially expressed genes (DEGs), with the x-axis representing log_2_ fold change and the y-axis representing −log_10_ false discovery rate (FDR). (**c**,**d**) Heatmaps illustrating normalized gene expression levels [log_10_(FPKM + 0.000001)], color-coded according to the scale bar.

Differential expression analysis revealed substantial transcriptional reprogramming under both stress conditions. In the HT-LH group, 464 genes were upregulated and 565 downregulated relative to the control (Figure 2a). In contrast, the HT-HH treatment induced a markedly stronger response, with 1145 upregulated and 1166 downregulated genes (Figure 2b). The MA plots further illustrate the distribution of differentially expressed genes, with red and blue dots indicating upregulated and downregulated genes, respectively (Figure S1). Hierarchical clustering demonstrated high consistency among biological replicates, confirming the robustness of treatment-specific expression patterns (Figure 2c,d).

### 2.3. Functional Annotation and Enrichment Analysis of DEGs

**Figure 3:**
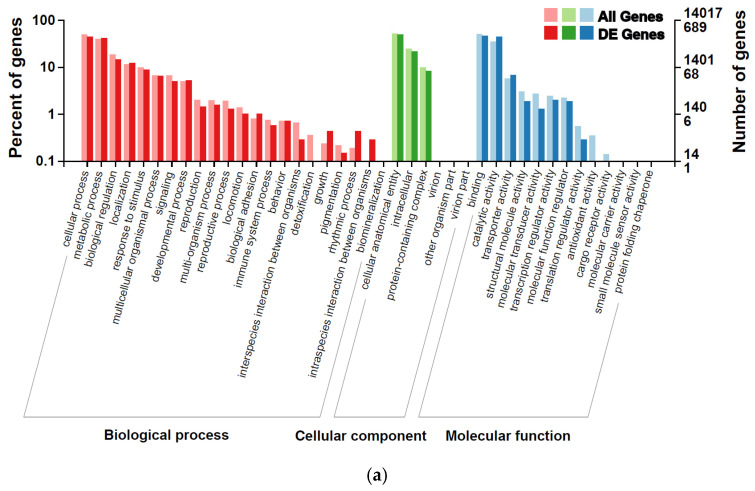

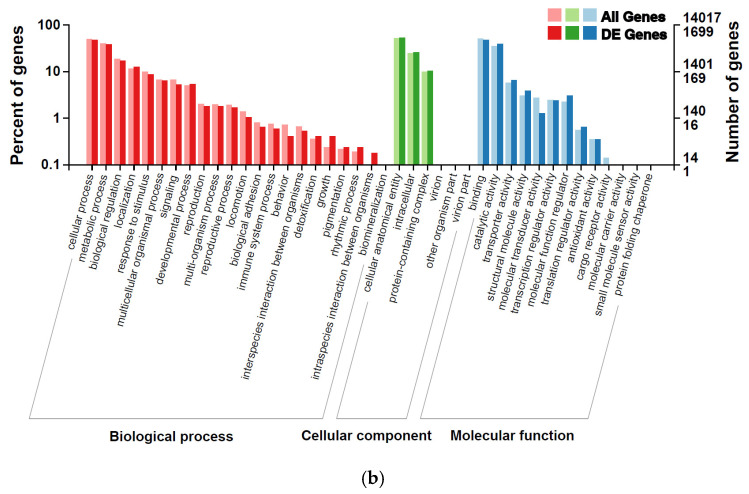
Gene Ontology (GO) enrichment analysis of total genes and differentially expressed genes (DEGs) in the tomato pinworm *T. absoluta* exposed to thermo-hygrometric stress. GO enrichment of total genes and DEGs in the HT-LH (40 °C, 50% RH) (**a**) and HT-HH (40 °C, 75% RH) (**b**) treatment groups. The x-axis indicates GO functional categories, the left y-axis represents the percentage of genes within each category, and the right y-axis shows the total number of annotated genes and DEGs.

**Figure 4:**
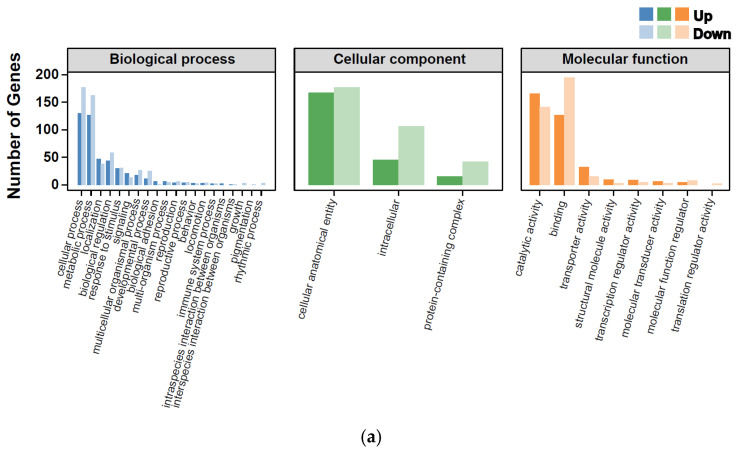

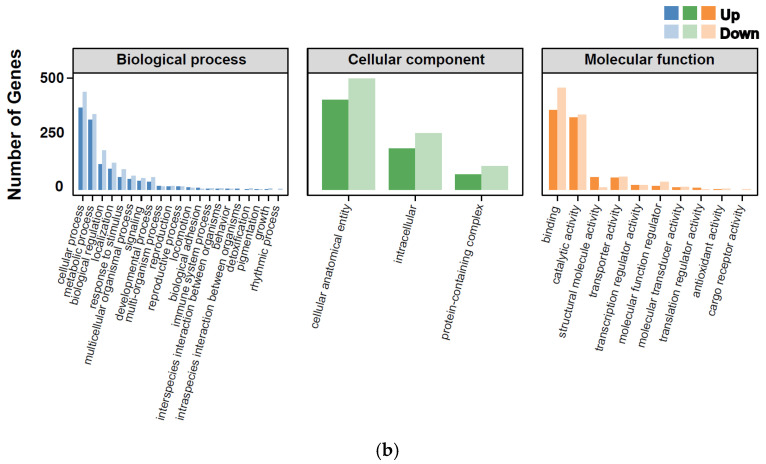
Gene Ontology (GO) enrichment analysis of up- and down-regulated genes in *T. absoluta* under combined temperature and humidity stress. GO enrichment of significantly up- and down-regulated DEGs in the HT-LH (**a**) and HT-HH (**b**) groups relative to the control. The x-axis represents GO terms, while the y-axis indicates both the number of annotated DEGs and their percentage within the DEG set.

Gene Ontology (GO) enrichment analysis revealed broad functional shifts associated with thermal and humidity stress (Figure 3 and Figure 4). In both HT-LH and HT-HH treatments, DEGs were predominantly enriched in biological processes related to cellular processes, metabolic processes, and biological regulation (Figure 3a,b). At the cellular component level, genes associated with intracellular structures, cellular anatomical entities, and protein complexes were overrepresented, while molecular function categories were dominated by binding, catalytic activity, and transporter activity. Additionally, a summary of KOG classifications of DEGs in the HT-LH and HT-HH treatments revealed functional preferences, such as metabolic functions and physiological biases, which are reflected in the different experimental groups (Figure S2).

**Figure 5:**
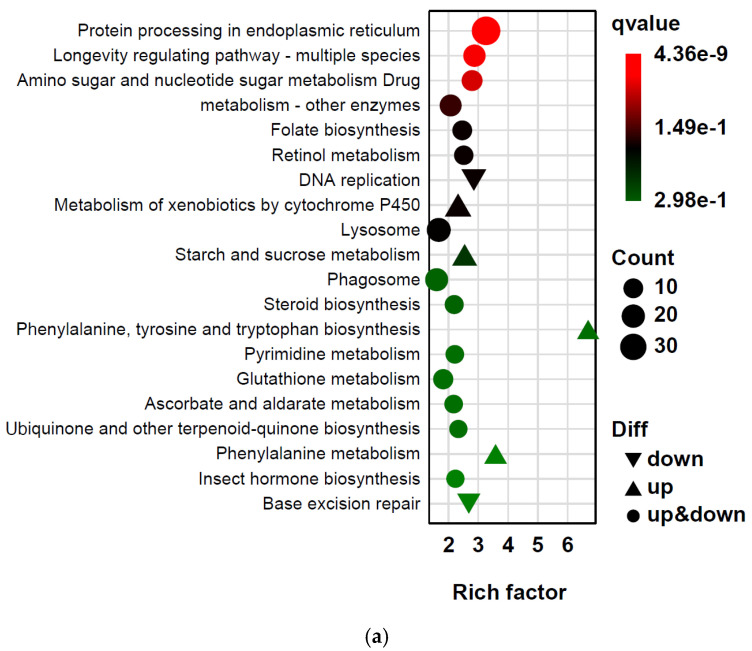

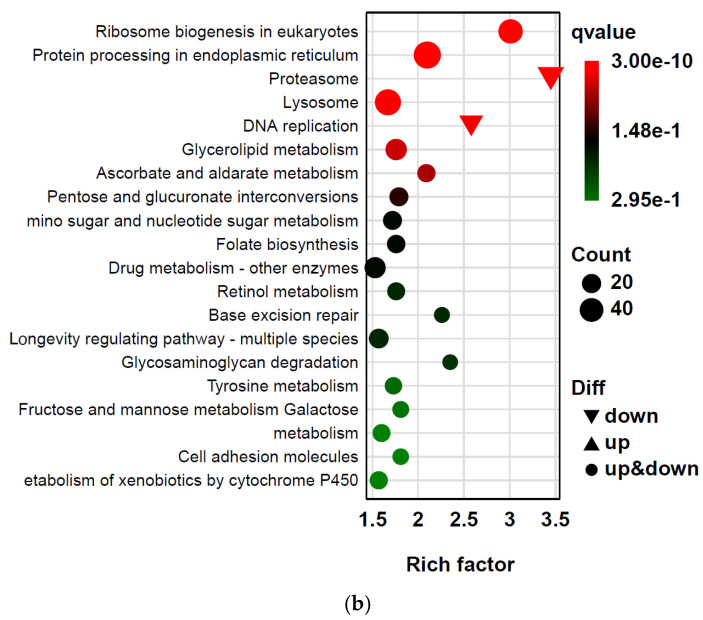
KEGG pathway enrichment analysis of differentially expressed genes (DEGs) in the tomato pinworm *T. absoluta* exposed to thermo-hygrometric stress. KEGG pathway enrichment of DEGs identified in the HT-LH (**a**) and HT-HH (**b**) treatment groups compared with the control. Each dot represents an enriched pathway; the y-axis lists KEGG pathways, and the x-axis shows the enrichment factor, calculated as the ratio of DEG annotation frequency relative to background gene annotation frequency.

KEGG pathway analysis further indicated stress-induced metabolic and cellular restructuring (Figure 5). Under HT-LH conditions, significantly enriched pathways included protein processing in the endoplasmic reticulum, longevity-regulating pathways, amino sugar and nucleotide sugar metabolism, and xenobiotic metabolism by cytochrome P450 (Figure 5a). Under HT-HH conditions, enrichment was strongest for ribosome biogenesis, protein processing in the endoplasmic reticulum, and proteasome-related pathways (Figure 5b). Collectively, these patterns indicate pronounced metabolic reorganization and enhanced cellular maintenance under combined thermal and humidity stress.

### 2.4. Validation of P450 Gene Expression by RT-qPCR

**Figure 6:**
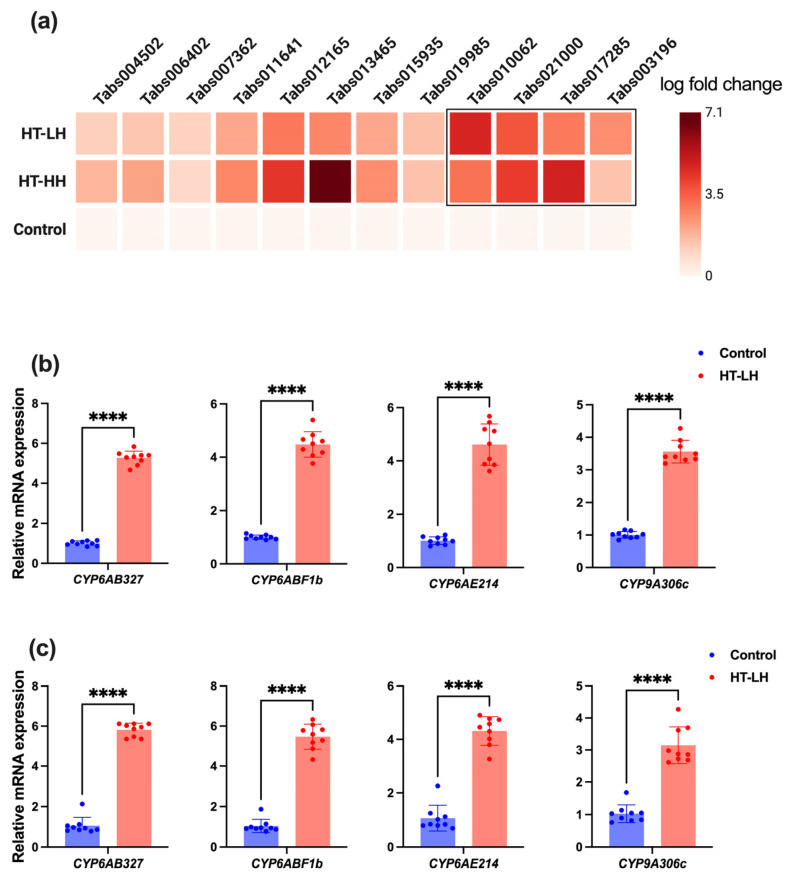
Log2FC (log fold change) of targeted genes in the transcriptome data, showing the relative expression changes between treatment and control (**a**). Relative expression of selected cytochrome P450 genes in the tomato pinworm *T. absoluta* under thermo-hygrometric stress. Relative mRNA expression levels of *CYP6AB327*, *CYP6ABF1b*, *CYP6AE214*, and *CYP9A306c* following exposure to HT-LH (**b**) and HT-HH (**c**) conditions, compared with the control group. Data are presented as mean ± SE of three independent biological replicates; **** indicates a statistically significant difference (*p* < 0.0001, Student’s *t*-test).

The targeted upregulated cytochrome P450 genes in both the HT-LH and HT-HH groups (Figure 6a) were validated by RT-qPCR analysis, which confirmed the transcriptomic results for four strongly induced genes, i.e., *CYP6AB327*, *CYP6ABF1b*, *CYP6AE214*, and *CYP9A306c*, further supporting the accuracy and reliability of the transcriptome data (Figure 6b,c). All four genes were significantly upregulated under both stress regimes. Following HT-LH exposure, transcript levels increased 5.27-, 4.47-, 4.61-, and 3.55-fold, respectively, relative to controls (*p* < 0.001 for all comparisons; Figure 6b). Even higher expression levels were observed under HT-HH, with fold changes of 5.82, 5.47, 4.31, and 3.14, respectively (*p* < 0.001; Figure 6c). These results indicate a consistent and humidity-dependent enhancement of P450 expression under high-temperature stress.

### 2.5. Phylogenetic Relationships and Conserved Motif Architecture of P450 Genes

**Figure 7:**
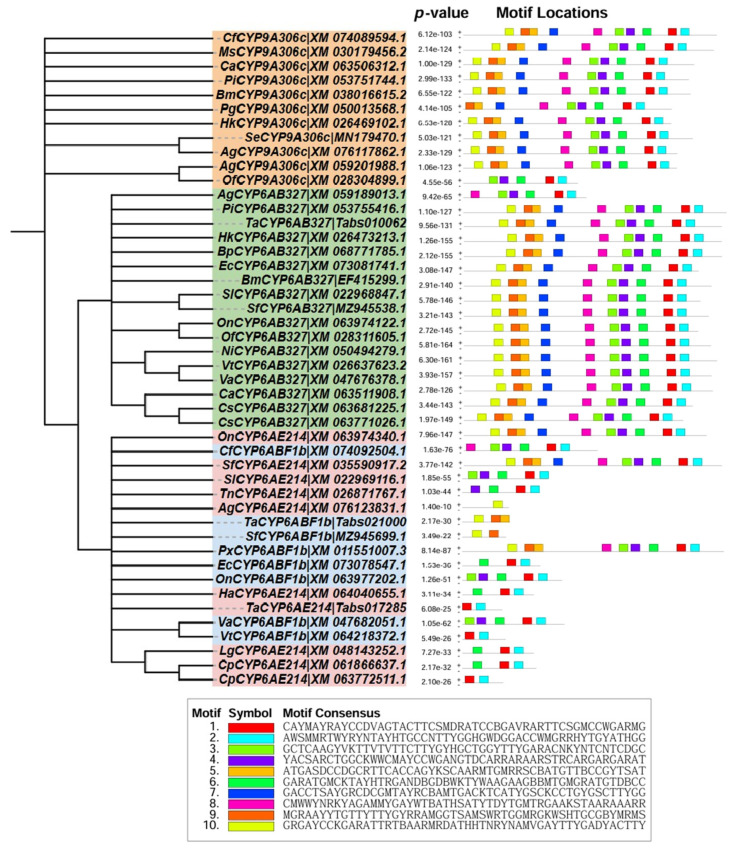
Phylogenetic relationships and conserved motif architecture of cytochrome P450 orthologs across representative insect species.

Phylogenetic analysis revealed clear clustering of the target genes within the *CYP6B* and *CYP9A* subfamilies (Figure 7). *CYP9A* genes clustered with orthologs from *Pectinophora gossypiella*, *Ostrinia nubilalis*, and *Spodoptera* spp., whereas *CYP6B* genes grouped with sequences from *Bombyx mori*, *Anticarsia gemmatalis*, and *T. absoluta*.

Motif analysis demonstrated highly conserved motif architectures within each subfamily, with all detected motifs showing strong statistical support (low *p*-values) (Figure 7). Several motifs were consistently positioned across orthologs, notably motif 1 and motif 6, suggesting conserved functional roles. The recurrence of motifs 2 and 5 within *CYP6B* genes further points to their potential involvement in enzymatic activity and stress adaptation. The conservation of these motifs across taxa underscores the functional importance of these P450 enzymes in insect metabolic and detoxification pathways.

### 2.6. Functional Validation of P450 Genes Using Nanocarrier-Mediated RNAi

**Figure 8:**
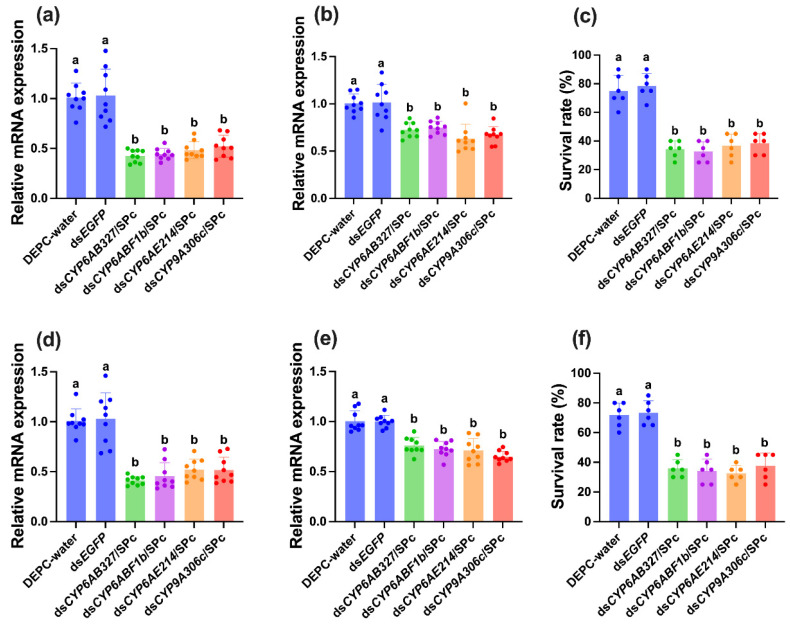
Functional validation of P450 genes in *Tuta absoluta* using nanocarrier-mediated RNA interference under thermo-hygrometric stress. Larvae were subjected to RNAi targeting four P450 genes (*CYP6AB327*, *CYP6ABF1b*, *CYP6AE214*, and *CYP9A306c*) and then exposed to HT-LH and HT-HH conditions. In panels (**a**–**c**), representing the HT-LH group, (**a**) shows the knockdown efficiency of each dsRNA treatment, with DEPC-water and ds*EGFP* as negative and non-target dsRNA controls; for each group, relative expression was measured for the corresponding target gene. Panel (**b**) shows the relative gene expression of these targets following HT-LH exposure, and (**c**) depicts larval survival under HT-LH stress. Panels (**d**–**f**) represent the HT-HH group, where (**d**) shows gene expression after RNAi for the four targets with the same controls, (**e**) shows relative expression following HT-HH exposure, and (**f**) presents survival rates under HT-HH conditions. Data were analyzed using one-way ANOVA followed by Tukey’s post hoc test (IBM SPSS Statistics v29), with different lowercase letters indicating statistically significant differences (*p* < 0.05).

Based on RNA-seq and RT-qPCR results, *CYP6AB327*, *CYP6ABF1b*, *CYP6AE214*, and *CYP9A306c* were selected for functional analysis using nanocarrier-mediated RNA interference (RNAi). Targeted knockdown of each gene significantly reduced transcript levels and larval survival under both stress regimes (Figure 8).

Under HT-LH conditions, RNAi treatment reduced gene expression to 0.42-, 0.44-, 0.48-, and 0.52-fold, respectively, relative to controls (F_5,53_ = 37.494, *p* < 0.001; Figure 8a). Following HT-LH exposure, partial transcript recovery was observed, but expression remained significantly suppressed (Figure 8b). Survival rates declined sharply to 32.5–38.3%, compared with control groups (F_5,35_ = 214.356, *p* < 0.001; Figure 8c).

A comparable pattern was observed under HT-HH conditions. RNAi reduced expression to 0.41–0.52-fold, with modest recovery after stress exposure but sustained suppression relative to controls (Figure 8d,e). Survival rates fell to 32.5–37.5% across RNAi treatments (F_5,35_ = 40.426, *p* < 0.001; Figure 8f). Figure S3 illustrates a conceptual model proposing the roles of P450 genes in thermo-hygrometric stress tolerance and suggests how their protective mechanisms may be disrupted following nanocarrier-mediated RNAi treatment. A conceptual model illustrating the proposed roles of P450 genes in thermo-hygrometric stress tolerance and how their protective mechanisms may be disrupted following nanocarrier-mediated RNAi treatment is shown in Figure S3. Together, these results provide direct functional evidence that P450 genes are essential for larval survival under high-temperature stress across contrasting humidity regimes.

## 3. Discussion

Temperature and humidity are critical abiotic factors governing insect survival, development, and population dynamics. In the present study, exposure to high temperatures significantly reduced the survival of third-instar tomato pinworm larvae, with mortality further intensified under both high- and low-humidity conditions. These results are consistent with studies reporting strong lethal effects of temperatures exceeding 37 °C across multiple life stages of the tomato pinworm [33,34].

The elevated mortality observed under combined heat–humidity stress likely reflects interactive physiological constraints, including impaired thermoregulation and water balance [35]. High humidity can limit evaporative cooling and reduce respiratory efficiency, thereby exacerbating thermal stress at higher temperatures [36]. Comparable responses have been documented in other lepidopteran pests, where elevated humidity amplifies heat-induced mortality through effects on cuticular permeability and gas exchange [36]. Together, these findings suggest that temperature-humidity interactions, rather than temperature alone, play a critical role in shaping pest performance under extreme climatic conditions. Importantly, a fraction of larvae survived even under severe stress, underscoring the considerable thermal tolerance of the tomato pinworm, a trait closely associated with its invasive success across diverse agro-climatic regions [10,34].

The transcriptomic analyses revealed robust and reproducible sequencing quality across all treatments. Exposure to combined thermal and humidity stress triggered extensive transcriptional reprogramming, with a substantial number of genes up- and downregulated under both HT-LH and HT-HH conditions, with the response more pronounced under HT-HH. The markedly larger DEG repertoire under HT-HH conditions is consistent with a higher physiological burden and indicates a stress-intensity-dependent molecular response. Such large-scale transcriptional plasticity suggests that the pinworm mobilizes a coordinated suite of stress-mitigation pathways to cope with simultaneous thermal and hygrometric challenges. Insects generally respond to thermal extremes through phenotypic plasticity and genetic regulation [37,38]. Functional annotation of DEGs in this study revealed an enrichment of fundamental biological processes, including cellular metabolism, biological regulation, and intracellular organization, reflecting broad physiological reprogramming under stress. This pattern is characteristic of insect responses to abiotic stress, where metabolic adjustment and cellular maintenance are central to survival [23,26].

Distinct pathway signatures merged between humidity regimes. Under HT-LH conditions, enrichment of xenobiotic and drug metabolism pathways, including cytochrome P450-associated processes, suggests enhanced detoxification activity, likely linked to mitigation of oxidative stress [39]. In contrast, HT-HH exposure prominently enriched pathways related to ribosome biogenesis, endoplasmic reticulum protein processing, and proteasome function, indicating a strong proteostasis response supporting protein synthesis, folding, and turnover [40]. These differences imply that the tomato pinworm deploys alternative, but overlapping, molecular strategies depending on the specific thermo-hygrometric context, highlighting its adaptive plasticity.

Cytochrome P450 enzymes are well known for their roles in detoxification, redox balance, and hormone metabolism [24,25], and members of the *CYP6* and *CYP9* families are particularly responsive to environmental stressors in insects [41,42]. Their coordinated upregulation observed here supports the notion of a network-level metabolic response rather than isolated gene effects [24]. However, sustained activation of these pathways is energetically costly and may impose fitness trade-offs, potentially contributing to reduced survival under prolonged or intensified stress. Similar patterns have been reported in other insect pests exposed to thermal extremes [26,41].

RT-qPCR validation confirmed that cytochrome P450 monooxygenases play a central role in the tomato pinworm’s response to combined temperature and humidity stress. All four genes (*CYP6AB327*, *CYP6ABF1b, CYP6AE214*, and *CYP9A306c*) were significantly upregulated under both HT-LH and HT-HH conditions, consistent with the RNA-seq results. The stronger induction observed under HT-HH exposure suggests that elevated humidity intensifies the physiological demand imposed by heat stress, thereby increasing reliance on metabolic and detoxification pathways [22,25]. Such responses have been observed in other lepidopterans, where CYP450 genes play important roles in thermotolerance [23,43]. Mortality rates were higher under HT-HH conditions than under HT-LH, highlighting the role of humidity in amplifying heat stress. Elevated moisture levels disrupt osmotic regulation, heighten metabolic demands, and modify cuticular permeability. Research has demonstrated that humidity affects both thermal tolerance and water-loss processes, thereby intensifying stress when protective systems are impaired [36,44].

Nanocarrier-assisted RNAi has previously been shown to enhance dsRNA stability and delivery efficiency in lepidopteran insects [31,32]. The sustained knockdown achieved in this study following oral delivery further highlights the utility of this approach for functional genomics. Functional validation using nanocarrier-mediated RNA interference demonstrated that targeted silencing of these P450 genes significantly reduced transcript abundance and was consistently associated with marked declines in larval survival under both humidity regimes. The absence of differences between dsEGFP and DEPC-water controls confirms that the observed effects were gene-specific. These results provide direct functional evidence that P450-mediated metabolic plasticity is a key determinant of stress tolerance in the tomato pinworm. Knockdown of these P450 genes led to lower transcript levels, impaired recovery after stress, and reduced insect survival, confirming their direct role in stress adaptation. Similar RNAi studies in other insects (AhCYP) and mites (*TtCYP3A2* and *TtCYP4V2*) have shown that silencing P450 genes reduces target survival under high temperatures [22,45]. Comparable findings have been reported in other insects, where knockout of the *BtCYP4C1* gene in *Bemisia tabaci* suppressed target gene expression, markedly reduced heat tolerance, and lowered survival at elevated temperatures [25], while dsRNA treatment of *BmCncC* in *Bombyx mori* cells significantly downregulated *CYP302A1* and *CYP306A1* transcription [46], collectively indicating that p450 genes contribute to high-temperature stress responses. The decreased survival after dsP450 gene treatment in *T. absoluta* aligns with these findings and supports the conserved function of chaperone-mediated defenses. From an applied perspective, these findings support the concept that disrupting adaptive stress-response pathways may increase pest vulnerability under extreme climatic conditions, although translation to field-level management will require further evaluation.

However, this study has limitations. Gene expression changes were assessed at the transcript level, and direct measurements of protein abundance, enzyme activity, oxidative stress markers, or cuticular traits were not conducted. In addition, experiments were performed under acute, constant stress conditions, whereas natural environments are characterized by fluctuating temperatures and humidity. Future studies integrating physiological, biochemical, and field-based approaches will be essential to fully resolve the mechanisms and ecological relevance of thermo-hygrometric adaptation in the tomato pinworm *T. absoluta*.

## 4. Materials and Methods

### 4.1. Insect Rearing

A laboratory colony of the South American tomato pinworm, *T. absoluta*, was established from individuals collected earlier on tomato plants in Yuxi, Yunnan Province, China, in June 2018. This colony was maintained over several generations without exposure to insecticides or other stress factors. The insects were reared on fresh tomato plants within transparent cages (30 × 30 × 30 cm), under controlled conditions: 25 ± 1 °C, 60 ± 5% relative humidity, and a 16:8 h light/dark cycle.

### 4.2. Exposure to Temperature and Humidity Stresses

To assess the impacts of temperature and humidity stress, 3rd instar larvae were subjected to high temperature combined with either low humidity (HT-LH: 40 °C, 50% RH) or high humidity (HT–HH: 40 °C, 75% RH) for 8 h. To produce synchronized cohorts, adults laid eggs on tomato plants for 12 h; then, the egg-bearing plants were moved to separate rearing cages.

Fresh tomato leaves were placed in Petri dishes lined with filter paper, with petioles wrapped in moist cotton to prevent desiccation. Each treatment had six biological replicates, each containing 20 larvae. The Petri dishes were transferred to incubators set to specific thermo-hygrometric conditions. After exposure, larvae were given a 1 h recovery period under standard laboratory conditions before assessing survival. Larvae were deemed dead if they did not respond to gentle prodding with a fine brush. Control larvae were kept under standard laboratory conditions. Surviving larvae were collected for subsequent molecular analyses.

### 4.3. RNA Extraction and Library Preparation

Total RNA was extracted from surviving third-instar larvae subjected to HT–LH, HT–HH, and control conditions using TRIzol Reagent (Life Technologies, Carlsbad, CA, USA). The RNA’s concentration and purity were measured with a NanoDrop 2000 spectrophotometer (Thermo Fisher Scientific, Wilmington, DE, USA), while its integrity was checked using an Agilent Bioanalyzer 2100 system (Agilent Technologies, Santa Clara, CA, USA). RNA-seq libraries were prepared with the NEBNext Ultra™ RNA Library Prep Kit for Illumina, following the manufacturer’s guidelines. In brief, mRNA was enriched using poly-T magnetic beads and then fragmented and reverse-transcribed to produce first- and second-strand cDNA. After end repair, adaptor ligation, and size selection (~240 bp) with AMPure XP beads, the libraries were PCR-amplified, purified, and assessed for quality via an Agilent Bioanalyzer 2100. Indexed libraries were clustered on a cBot Cluster Generation System with TruSeq PE Cluster Kit v4-cBot-HS (Illumina, San Diego, CA, USA) and sequenced on an Illumina platform to obtain paired-end reads.

### 4.4. Data Quality Control, Read Mapping, and Functional Annotation

Raw reads were filtered using custom Perl scripts to eliminate adapter sequences, poly-N reads, and low-quality data. The resulting clean reads were assessed using standard quality metrics such as Q20, Q30, GC content, and duplication rates. High-quality reads were then aligned to the *T. absoluta* reference genome from InsectBase 2.0 using HISAT2. For further analysis, only reads that matched perfectly or had at most one mismatch were kept.

### 4.5. Differential Gene Expression and Enrichment Analyses

Differential gene expression was examined with DESeq2, where *p*-values were adjusted for multiple comparisons via the Benjamini–Hochberg false discovery rate (FDR). Genes with adjusted *p*-values below 0.01 were deemed significantly differentially expressed. Further filtering of DEGs applied thresholds of |log_2_ fold change| ≥ 1 and *p* < 0.05. Functional enrichment was analyzed using the GOseq R package based on the Wallenius non-central hyper-geometric distribution for Gene Ontology (GO) terms and KOBAS for KEGG pathway analysis.

### 4.6. Phylogenetic and Motif Analysis

Homologous amino acid sequences of the targeted genes were obtained from publicly accessible insect databases through BLAST searches. Full-length sequences were aligned with ClustalW in MEGA11 software (version 11.0.13). Phylogenetic trees were generated using the maximum likelihood method with 1000 bootstrap replicates, employing pairwise deletion to handle gaps. Conserved motif analysis was conducted using MEME v5.5.7, and the motif architectures were visualized proportionally.

### 4.7. Reverse Transcription Quantitative PCR (RT-qPCR)

RT-qPCR was used to measure the expression levels of P450 genes (*CYP6AB327*, *CYP6ABF1b*, *CYP6AE214*, and *CYP9A306c*). Total RNA was extracted using the RNAsimple Total RNA Kit (Tiangen Biotechnology, Beijing, China) and reverse-transcribed from 1 µg of RNA using the iScript™ cDNA Synthesis Kit (Bio-Rad, Hercules CA, USA). The RT-qPCR reactions were conducted on a CFX Connect™ Real-Time PCR System (Bio-Rad, Hercules CA, USA) with SYBR Green chemistry. Expression levels were determined via the 2^−∆∆Ct^ method, using *EF1α* and *RPL28* as reference genes. Each treatment had three biological replicates, with each analyzed in triplicate. Primer sequences are listed in Table 2.

### 4.8. dsRNA Synthesis and Nanoparticle Complex Preparation

Double-stranded RNA was produced with the T7 RNAi Transcription Kit (Nanjing Vazyme Biotech Co., Ltd., Nanjing, China) according to the manufacturer’s instructions. Templates consisted of gene-specific PCR products with T7 promoter sequences at both ends. Following in vitro transcription, enzymatic removal of residual DNA and single-stranded RNA was performed. The purified dsRNA was dissolved in RNase-free water, and its concentration and quality were measured spectrophotometrically. dsRNA targeting EGFP served as a negative control. For nanoparticle formulation, dsRNA was combined with star polycation (SPc) at a 1:1 mass ratio (500 ng µL^−1^ each) and incubated at room temperature for 15 min to facilitate complex formation [31]. All dsRNA/SPc complexes were stored at −20 °C until needed.

### 4.9. Nanocarrier-Mediated RNA Interference (RNAi)

Fresh tomato leaves were sprayed with dsRNA/SPc complexes targeting specific P450 genes or EGFP at 500 ng µL^−1^. After air-drying, they were placed in Petri dishes with moist filter paper. Each treatment had three replicates of 20 third-instar larvae. Control groups included DEPC-treated leaves and dsEGFP/SPc. After 48 h of feeding, surviving larvae were collected for RNA extraction and RT-qPCR to evaluate gene silencing. Subsequently, larvae underwent an additional 8 h stress period of either HT–LH or HT–HH, followed by reassessment of survival rates and gene expression.

### 4.10. Data Analysis

Survival and RT-qPCR data were analyzed with SPSS v29. Group comparisons were performed using Student’s *t*-tests or one-way ANOVA, followed by Tukey’s post hoc test. Significance was set at *p* < 0.05. Graphs were created using GraphPad Prism 9, and transcriptomic visualizations were generated on the BMK Cloud Platform.

## 5. Conclusions

Overall, our findings demonstrate that while high temperatures combined with contrasting humidity regimes reduce tomato pinworm survival, this species possesses robust molecular mechanisms that confer partial stress tolerance. The stronger transcriptional response under HT-HH conditions suggests that tolerance is limited, but not eliminated, under severe stress. In the context of climate change, these results highlight the need to consider interacting abiotic drivers when predicting pest dynamics and point to adaptive metabolic pathways as leverage for sustainable pest management. Moreover, our findings advance understanding of the molecular basis of tomato pinworm ecological success and highlight nanocarrier-assisted RNAi as both a powerful research tool and a potential avenue for next-generation pest management.

## Figures and Tables

**Table 1:** Summary statistics of high-throughput sequencing data generated from the tomato pinworm *T. absoluta* exposed to high temperature (40 °C) under low (50% RH) and high (75% RH) humidity conditions, compared with the control group.

Samples	Clean Reads	Clean Bases	GC Content (%)	Q30 (%)
Control-1	27,869,743	8,320,883,113	46.05%	98.93%
Control-2	21,722,356	6,498,227,930	46.90%	97.87%
Control-3	23,147,705	6,908,892,628	46.62%	98.90%
T40/50-1	20,459,875	6,120,302,772	46.60%	97.94%
T40/50-2	20,731,894	6,198,674,150	46.91%	98.79%
T40/50-3	20,789,209	6,218,446,877	47.54%	98.23%
T40/75-1	27,699,372	8,278,935,932	47.94%	98.89%
T40/75-2	19,940,020	5,967,451,568	46.58%	98.79%
T40/75-3	19,319,693	5,782,670,963	47.35%	98.12%

**Table 2:** Primer sequences used for RT-qPCR and dsRNA validation and dsRNA synthesis targeting P450 genes in the tomato pinworm *T. absoluta*.

Primer Name	Forward Sequence	Reverse Sequence
*CYP6AB327*	ACACTTTTCGCTGCACAAGT	ACACTTTTCGCTGCACAAGT
*CYP6ABF1b*	GATGGCAGCATTCGTGTCTT	AGCTGAAGTGAATGCCCTCT
*CYP6AE214*	ACCTCTGCCTTTCTTCGGAA	TGTACGACTCCCTGGGAAAC
*CYP9A306c*	CGAGGTGAAAATCATGGCGT	CAGTGTCCACCCTTCATCCT
*RPL28*	TCAGACGTGCTGAACACACA	GCCAGTCTTGGACAACCATT
*TaEF1α*	GAAGCCTGGTATGGTTGTCGT	GGGTGGGTTGTTCTTTGTG
*dsEGFP*	TAATACGACTCACTATAGGGAAGTTCAGCGTGTCCGGCGAGG	TAATACGACTCACTATAGGGCACCTTGATGCCGTTCTTCTGC
*dsCYP6AB327*	TAATACGACTCACTATAGGGATCGTCCTAGCGCTTTGTGT	TAATACGACTCACTATAGGGATTCAGCCCTCGCAGATAGA
*dsCYP6ABF1b*	TAATACGACTCACTATAGGGAGCAGTTTCCAAAAGAGCCA	TAATACGACTCACTATAGGGCATGGTATGAAACACGCTCG
*dsCYP6AE214*	TAATACGACTCACTATAGGGGTTTCCCAGGGAGTCGTACA	TAATACGACTCACTATAGGGAACCAACCGAGCCAATACAG
*dsCYP9A306c*	TAATACGACTCACTATAGGGTCCTTCTTCACGAGTTGGCT	TAATACGACTCACTATAGGGCGCTCAGGGTCAAACTTCTC

The underline letters indicated the T7 promoter sequence.

## Data Availability

The authors confirm that the data supporting the findings of this study are available in the article.

## Supplementary Materials

The following supporting information can be downloaded at: https://www.mdpi.com/article/10.3390/ijms27072935/s1.

## References

[1] Raihan A. (2023). A review of the global climate change impacts, adaptation strategies, mitigations options in the socio-economic and environmental sectors. J. Environ. Sci. Econ..

[2] Foster P.M., Smith C., Walsh T., Johnson R., Anderson M., Lee H., Brown L., Davis K., White G., Green J. (2024). Indicators of Global Climate Change 2023: Annual Update of Key Indicators of the State of the Climate System and Human Influence. Earth Syst. Sci. Data.

[3] Gyuleva G., Knutti R., Sippel S. (2025). Combination of internal variability and forced response reconciles observed 2023–2024 warming. Geophys. Res. Lett..

[4] Coelho M.T.P., Barreto E., Rangel T.F., Diniz-Filho J.Á., Wuest R.O., Bach W., Skeels A., McFadden I.R., Roberts D.W., Pellissier L. (2023). The geography of climate and the global patterns of species diversity. Nature.

[5] Murtaza G., Ullah F., Zhao Z., Liao Z., Trujillo-Pahua V., Ramirez-Romero R., Li Z. (2025). Insect Responses to Heatwaves and Their Influence on Integrated Pest Management. Entomol. Gen..

[6] Mo Y., Yuan Z., Han Q., Cai F., Chen X., Liu J., Yi T., Yang Z. (2026). Survival and fitness responses of *Diaphorina citri* to short-term freezing stress. Entomol. Gen..

[7] Hill M.P., Clusella-trullas S., Terblanche J.S., Richardson D.M. (2016). Drivers, impacts, mechanisms and adaptation in insect invasions. Biol. Invasions.

[8] Bradshaw C.J.A., Leroy B., Bellard C., Roiz D., Albert C., Fournier A., Barbet-Massin M., Salles J.-M., Simard F., Courchamp F. (2016). Massive Yet Grossly Underestimated Global Costs of Invasive Insects. Nat. Commun..

[9] Campos M.R., Biondi A., Adiga A., Guedes R.N.C., Desneux N. (2017). From the Western Palaerarctic region to beyond: *Tuta absoluta* 10 years after invading Europe. J. Pest Sci..

[10] Biondi A., Guedes R.N.C., Wan F.H., Desneux N. (2018). Ecology, worldwide spread, and management of the invasive South American tomato pinworm, *Tuta absoluta*: Past, present, and future. Annu. Rev. Entomol..

[11] Peng H., Zhang Y., Arnó J., Mansour R. (2024). Research toward enhancing integrated management of Tuta absoluta, an ongoing invasive threat in Afro-Eurasia. Entomol. Gen..

[12] Desneux N., Wajnberg E., Wyckhuys K.A., Burgio G., Arpaia S., Narváez-Vasquez C.A., González-Cabrera J., Catalán Ruescas D., Tabone E., Frandon J. (2010). Biological invasion of European tomato crops by Tuta absoluta: Ecology, geographic expansion and prospects for biological control. J. Pest Sci..

[13] Zhang G., Xian X., Zhang Y., Liu W., Liu H., Feng X., Ma D., Wang Y., Gao Y., Zhang R. (2021). Outbreak of the South American tomato leafminer, *Tuta absoluta*, in the Chinese mainland: Geographic and potential host range expansion. Pest Manag. Sci..

[14] Desneux N., Luna M.G., Guillemaud T., Urbaneja A. (2011). The invasive South American tomato pinworm, *Tuta absoluta*, continues to spread in Afro-Eurasia and beyond: The new threat to tomato world production. J. Pest Sci..

[15] Guillemaud T., Blin A., Le Goff I., Desneux N., Reyes M., Tabone E., Tsagkarakou A., Nono L., Lombart E. (2015). The tomato borer, *Tuta absoluta*, invading the Mediterranean Basin, originates from a single introduction from Central Chile. Sci. Rep..

[16] Azrag A.G.A., Obala F., Tonnang H.E.Z., Hogg B.N., Ndela S., Mohamed S.A. (2023). Predicting the impact of climate change on the potential distribution of the invasive tomato pinworm *Phthorimaea absoluta* (Meyrick) (Lepidoptera: Gelechiidae). Sci. Rep..

[17] Campos M.R., Béarez P., Amiens-Desneux E., Ponti L., Gutierrez A.P., Biondi A., Adiga A., Desneux N. (2021). Thermal biology of *Tuta absoluta*: Demographic parameters and facultative diapause. J. Pest Sci..

[18] Machekano H., Mutamiswa R., Nyamukondiwa C. (2018). Evidence of rapid spread and establishment of *Tuta Absoluta* (Meyrick) (Lepidoptera: Gelechiidae) in Semi-Arid Botswana. Agric. Food Secur..

[19] Wang X.D., Lin Z.K., Ji S.X., Bi S.Y., Liu W.X., Zhang G.F., Wan F.H., Lü Z.C. (2021). Molecular characterization of TRPA subfamily genes and function in temperature preference in *Tuta absoluta* (Meyrick) (Lepidoptera: Gelechiidae). Int. J. Mol. Sci..

[20] Tao Y.D., Liu Y., Wan X.S., Xu J., Fu D.Y., Zhang J.Z. (2023). High and low temperatures differentially affect survival, reproduction, and gene transcription in male and female moths of *Spodoptera frugiperda*. Insects.

[21] Cui Z., Gao Z., Li Y., Sun J., Mang D. (2025). Heat-sensing receptor pathway promotes thermal tolerance via chitin remodeling in an invasive lepidopteran pest. Entomol. Gen..

[22] Zhang H., Zhao M., Liu Y., Zhou Z., Guo J. (2018). Identification of cytochrome P450 monooxygenase genes and their expression in response to high temperature in the alligatorweed flea beetle *Agasicles hygrophila* (Coleoptera: Chrysomelidae). Sci. Rep..

[23] Liu Y., Su H., Li R., Li X., Xu Y., Dai X., Zhou Y., Wang H. (2017). Comparative transcriptome analysis of *Glyphodes pyloalis* Walker (Lepidoptera: Pyralidae) reveals novel insights into heat stress tolerance in insects. BMC Genom..

[24] Wang Y.C., Chang Y.W., Bai J., Zhang X.X., Iqbal J., Lu M.X., Hu J., Du Y.Z. (2021). High temperature stress induces expression of CYP450 genes and contributes to insecticide tolerance in *Liriomyza trifolii*. Pestic. Biochem. Physiol..

[25] Shen X., Liu W., Wan F., Lv Z., Guo J. (2021). The Role of Cytochrome P450 4C1 and Carbonic Anhydrase 3 in Response to Temperature Stress in *Bemisia tabaci*. Insects.

[26] Vatanparast M., Park Y. (2022). Differential transcriptome analysis reveals genes related to low- and high-temperature stress in the fall armyworm, *Spodoptera frugiperda*. Front. Physiol..

[27] Qiu Y., Tittiger C., Wicker-Thomas C., Le Goff G., Young S., Wajnberg E., Fricaux T., Taquet N., Blomquist G.J., Feyereisen R. (2012). An insect-specific P450 oxidative decarbonylase for cuticular hydrocarbon biosynthesis. Proc. Natl. Acad. Sci. USA.

[28] Chen Z., Ren L., Li J., Fu N., Yun Q., Luo Y. (2024). Chromosomal-level genome assembly of *Hylurgus ligniperda*: Insights into host adaptation and environmental tolerance. BMC Genom..

[29] Burand J.P., Hunter W.B. (2013). RNAi: Future in insect management. J. Invertebr. Pathol..

[30] Yang W., Wang B., Lei G., Chen G., Liu D. (2022). Advances in nanocarriers to improve the stability of dsRNA in the environment. Front. Bioeng. Biotechnol..

[31] Yan S., Yin M.Z., Shen J. (2023). Nanoparticle-based nontransformative RNA insecticides for sustainable pest control: Mechanisms, current status and challenges. Entomol. Gen..

[32] Ullah F., G G.-P.-P., Gul H., Panda R.M., Murtaza G., Zhang Z., Huang J., Li X., Desneux N., Lu Y. (2025). Nanocarrier-mediated RNAi of *CYP9E2* and *CYB5R* enhance susceptibility of invasive tomato pest, *Tuta absoluta* to cyantraniliprole. Front. Plant Sci..

[33] Krechemer F.D.S., Foerster L.A. (2015). *Tuta absoluta* (Lepidoptera: Gelechiidae): Thermal requirements and effect of temperature on development, survival, reproduction and longevity. Eur. J. Entomol..

[34] Zhou J., Luo W., Song S., Wang Z., Zhu X., Gao S., He W., Xu J. (2024). The impact of high-temperature stress on the growth and development of *Tuta absoluta* (Meyrick). Insects.

[35] O’Donnell M.J. (2022). A perspective on insect water balance. J. Exp. Biol..

[36] Chown S.L., Sørensen J.G., Terblanche J.S. (2011). Water loss in insects: An environmental change perspective. J. Insect Physiol..

[37] Sgrò C.M., Terblanche J.S., Hoffmann A.A. (2016). What can plasticity contribute to insect responses to climate change?. Annu. Rev. Entomol..

[38] Karl I., Becker M., Hinzke T., Mielke M., Schiffler M., Fischer K. (2014). Interactive effects of acclimation temperature and short-term stress exposure on resistance traits in the butterfly *Bicyclus anynana*. Physiol. Entomol..

[39] Nauen R., Bass C., Feyereisen R., Vontas J. (2022). The role of cytochrome P450s in insect toxicology and resistance. Annu. Rev. Entomol..

[40] González-Tokman D., Córdoba-Aguilar A., Dáttilo W., Lira-Noriega A., Sánchez-Guillén R.A., Villalobos F. (2020). Insect responses to heat: Physiological mechanisms, evolution and ecological implications in a warming world. Biol. Rev..

[41] Wang Y.C., Chang Y.W., Xie H.F., Gong W.R., Wu C.D., Du Y.Z. (2024). The cytochrome P450 gene CYP4g1 driven by high temperature confers abamectin tolerance on *Liriomyza trifolii* through promoting cuticular hydrocarbons biosynthesis. Pestic. Biochem. Physiol..

[42] Dermauw W., Leeuwen T.V., Feyereisen R. (2020). Diversity and evolution of the P450 family in arthropods. Insect Biochem. Mol. Biol..

[43] Li B., Li M., Wu J., Xu X. (2019). Transcriptomic analysis of differentially expressed genes in the oriental armyworm *Mythimna separata* Walker at different temperatures. Comp. Biochem. Physiol. D Genom. Proteom..

[44] Riddell E.A., Mutanen M., Ghalambor C.K. (2023). Hydric effects on thermal tolerances influence climate vulnerability in a high-latitude beetle. Glob. Change Biol..

[45] Li W., Wu X., Hu T., Liu L., Wang S., Song L. (2023). The role of cytochrome P450 3A2 and 4V2 in response to high-temperature stress in *Tetranychus truncatus* (Acari: Tetranychidae). Exp. Appl. Acarol..

[46] Li J., Mao T., Wang H., Lu Z., Qu J. (2019). The CncC/keap1 pathway is activated in high temperature-induced metamorphosis and mediates the expression of *Cyp450* genes in silkworm, *Bombyx mori*. Biochem. Biophys. Res. Commun..

