# Supplementary material for: Cytochrome P450 Genes Mediate High-Temperature Adaptation Under Diverging Humidity Conditions in Tuta absoluta
Source: Int J Mol Sci. 2026 Mar 24;27(7):2935. doi: 10.3390/ijms27072935 (PMC13072793; doi:10.3390/ijms27072935)
Supplement: Supplementary file 1 [file ijms-27-02935-s001.zip › ijms-4166147-supplementary.pdf]

Supplementary files:

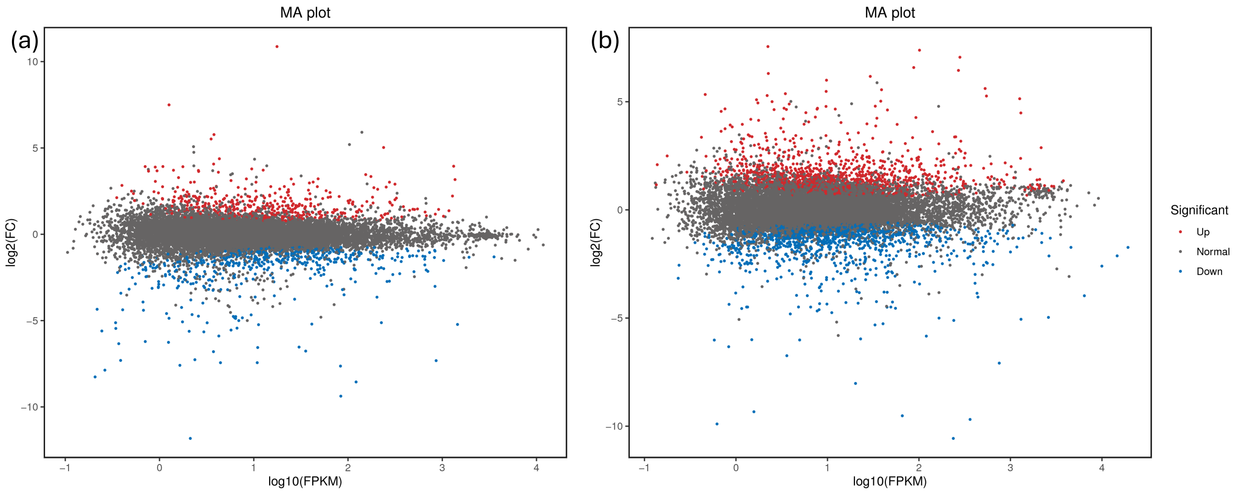

Figure S1. MA plot of differentially expressed genes in HT-LH (a) and HT-HH (b). Each dot represents a single gene. The dots coloured in red and green stand for significantly up-regulated and down-regulated genes, respectively. Black dots stand for the genes without a significant difference in expression between the two samples.

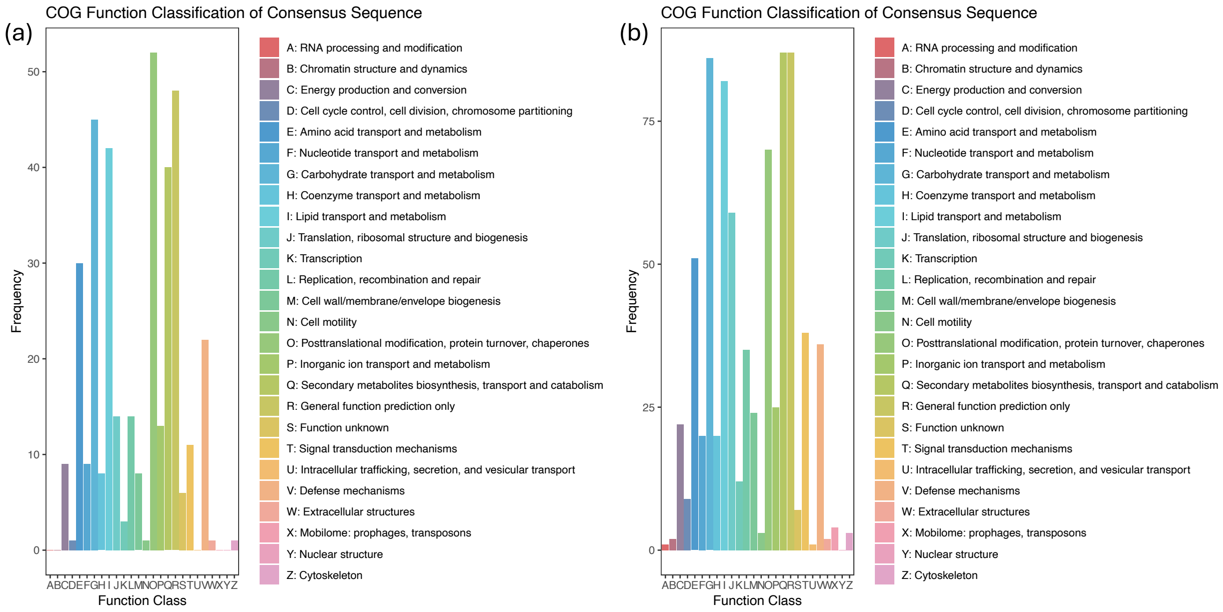

Figure S2. Summary of KOG classifications on DEGs in HT-LH (a) and HT-HH (b). In the different functional classes, the number of genes reflects the preference of gene functions in different experimental groups, such as metabolic function or physiological bias, etc.

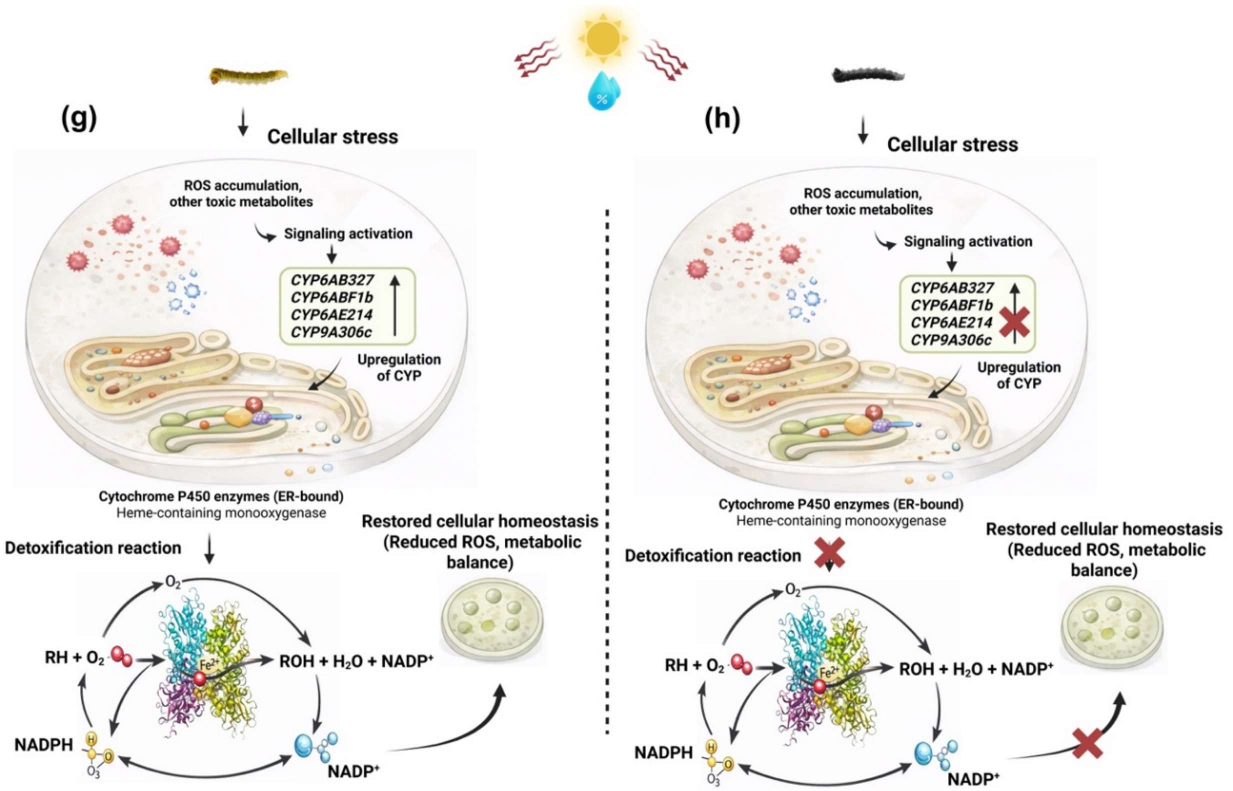

Figure S3. Conceptual model illustrating proposed roles of P450 genes in thermo-hygrometric stress tolerance and disruption of protective mechanisms following nanocarrier-mediated RNAi.
